# Peliminary exploration on the differential diagnosis between meningioma and schwannoma using contrast-enhanced T_1_WI flow-sensitive black-blood sequence

**DOI:** 10.3389/fonc.2022.1006190

**Published:** 2023-01-05

**Authors:** Xin Cao, Kun Lv, Siting Xu, Zhe Feng, Xuyang Yin, Lei Pan, Daoying Geng, Jun Zhang

**Affiliations:** ^1^ Department of Radiology, Huashan Hospital, Fudan University, Shanghai, China; ^2^ National Center for Neurological Disorders, Shanghai, China; ^3^ Center for Shanghai Intelligent Imaging for Critical Brain Diseases Engineering and Technology Reasearch, Shanghai, China

**Keywords:** flow-sensitive black blood, meningioma, schwannoma, intratumoral microbleeds, microbleed density index

## Abstract

**Introduction:**

Contrast-enhanced T_1_WI flow-sensitive black-blood (CE-T1WI FSBB) is a newly developed sequence which had not been widely used for differential diagnosis of brain tumors.

**Methods:**

To quantify the pre-operative imaging features of intratumoral microbleeds and intratumoral vessels using CE-T_1_WI FSBB scan and study the differences in biological behavior of meningiomas and schwannomas underlying the imaging features. Seventy-three cases of meningiomas and 24 cases of schwannomas confirmed by postoperative pathology were included. Two neuroradiologists independently counted intratumoral vessels and intratumoral microbleeds based on CE-T_1_WI FSBB images. The vessel density index (VDI) and microbleed density index (MDI) were the number of intratumoral vessels and the number of intratumoral microbleeds divided by the tumor volume, respectively. The consistency test of intratumoral vessel count and intratumoral microbleed count based on CE-T_1_WI FSBB were summarized using 2-way random intraclass correlation coefficients (ICC). Mann–Whitney U-test and chi-square test were used to determine significant differences between meningiomas and schwannomas, and fibrous meningiomas and epithelial meningiomas. *P*<0.05 was considered statistically significant.

**Results:**

The ICC of intratumoral vessels count and intratumoral microbleeds count were 0.89 and 0.99, respectively. There were significant differences in the number of intratumoral microbleeds (*P*<0.01) and MDI values (*P*<0.01) between meningiomas and schwannomas. There were no differences in the number of intratumoral vessels (*P*=0.64), VDI (*P*=0.17), or tumor volume (*P*=0.33). There were also differences in the number of intratumoral microbleeds (*P*<0.01), the MDI value (*P*<0.01), and the sex of patients (*P*<0.05) between fibrous meningiomas and epithelial meningiomas.

**Discussion:**

CE-T_1_WI FSBB can be a new technique for differentiating schwannomas from meningiomas, and even different types of meningiomas. Schwannomas have a higher incidence of intratumoral hemorrhage, more intratumoral microbleeds, and higher MDI values than meningiomas, which provides a new basis for preoperative differential diagnosis and treatment decisions.

## Introduction

Flow Sensitive Black Blood (FSBB) is a black-blood method composed of a spoiled gradient echo (SPGR) sequence combined with a motion probing gradient (MPG) pulse, where the flow-sensitive gradient can dephase blood signals with a wide range of flow velocities and an appropriate b value (1, 2). The FSBB sequence has the characteristics of high resolution and short scanning time, which can result in a higher visualizing capability of small blood vessels in the brain (3); On the other hand, FSBB sequence has the principle similar to T2 * weighted imaging, which can detect the intratumoral microbleeds accurately through the paramagnetic effect of hemosiderin deposition (4).

**Figure 1:**
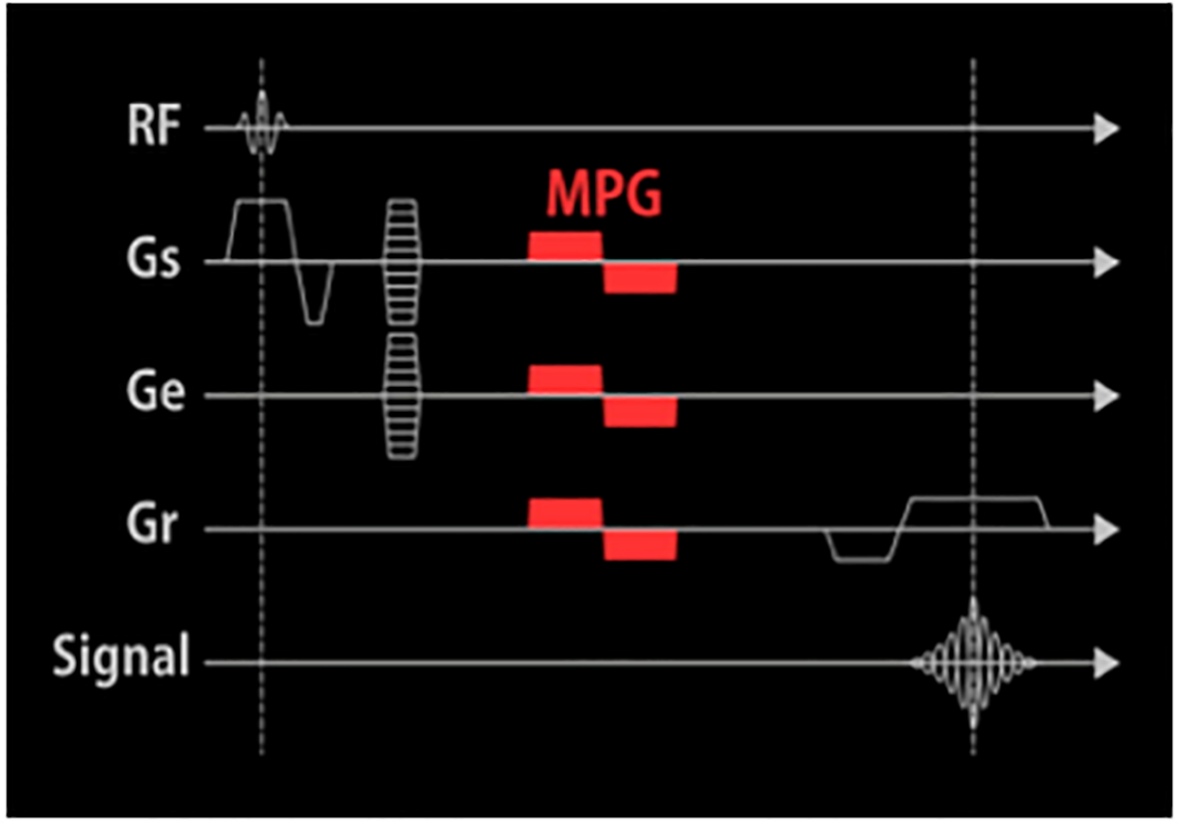
The pulse sequence diagram of CE-T_1_WI FSBB. An MPG pulse is applied at the end of the excitation RF pulse and the front of the Gro in three directions: Gss, Gpe, and Gro.

The 3D contrast-enhanced T1-weighted Imaging Flow Sensitive Black Blood (CE-T_1_WI FSBB), commonly known as “firefly imaging”, suppresses the flowing blood signal by the flow phase destroying the pulse, thus providing a clear contrast compared with the enhanced brain tumor with a high signal (**Figure 1**). The thickness of the scanning layer can reach 0.2 mm, and the spatial resolution of the image is greatly improved, which has advantages in displaying smaller vessels and microbleeds. Previous studies had shown that the intraclass correlation coefficients (ICC) of counting lenticulostriate artery branches based on FSBB was 0.89 (95% CI:0.73–0.96), which was higher than 0.78 (95% CI:0.45–0.91) of magnetic resonance angiography (MRA) (1). The length and quality of small vessel visualization were significantly better with FSBB scanning than with MRA, which was confirmed by histological results (5, 6). According to a recent study, the ICCs for intratumoral vessel count and intratumoral microbleed count on CE-T_1_WI FSBB were 0.92 and 0.99 (7).

Some studies have revealed that hemorrhagic lesions and blood vessels in brain tumors can reflect pathological classification and grade as well as molecular biomarkers (8–10). Brain tumors of different WHO grades and pathological classifications may have different densities of intratumoral vessels or microbleeds. Brain tumor pathological neovascularization is prone to blood-brain barrier damage, leading to secondary changes such as microbleeds and extensive necrosis (10). Intratumoral hemorrhage in vestibular schwannomas is a possible mechanism of hearing loss (11). Intratumoral hemosiderin and microvessel density were significantly positively correlated with the size and tumor growth index of vestibular schwannomas (12). Hemosiderin deposition density was significantly higher in cystic and inhomogeneous tumors than in homogeneous tumors, and microvessel density was significantly higher in tumors with a high number of CD68-positive cells (12). Although the T2*-weighted gradient-echo (GRE) sequence or 3D susceptibility-weighted imaging has been used to detect intratumoral microhemorrhages to help differentiate vestibular schwannoma from meningioma in the cerebellopontine angle (CPA) (13, 14), few studies have focused on accurately assessing the density of microbleeds in these benign tumors. This study aimed to quantify image features of intratumoral vessels and intratumoral microbleeds using the recently developed 3D CE-T_1_WI FSBB imaging technology and to study the differences in biological behavior of meningiomas and schwannomas underlying the imaging features.

## Materials and methods

### Study design and participants

This study was approved by the Institutional Review Board of Huashan Hospital, Fudan University. Written informed consent was obtained from patients or their legal representatives before enrollment in the study. Consecutive patients with extraparenchymal tumors were prospectively recruited between September 2020 and April 2022. Patient inclusion criteria were as follows: (I) extraparenchymal tumors detected using routine cranial MRI and CT; (II) meningioma or schwannoma confirmed by histopathology; and (III) completed CE-T1WI FSBB sequence scanning. The exclusion criteria were as follows: (I) contraindication to CE-MRI (such as ferromagnetic implants, pacemakers, allergy, etc.); (II) image quality or tumor visualization that did not meet the research standards; (III) too much bleeding in the tumor affects the display and counting of blood vessels; (IV) undergoing radiotherapy or chemotherapy; (V) recurrent tumors after surgery; and (VI) without postoperative histopathological findings.

### CE-T_1_WI FSBB protocol

All patients underwent cranial MRI scans with a 3.0 T MR system (Vantage Titan, Canon) using a 32-element phased-array head coil and receiver channels combined with parallel imaging capability. After localization, CE-T_1_WI FSBB images were obtained using the following parameters: axial orientation, echo time (TE) = 20.0 ms, repetition time (TR) = 29.0 ms, field of view (FOV) = 230 mm×230 mm, matrix size = 256×256, slice thickness = 3.0 mm, number of slices = 40, reconstruction thickness = 1.5 mm, reconstruction layers = 80, voxel = 0.89×0.89×1.50 mm^3^, flip angle = 26°, and scan time = 2 min13 s.

*Image analysis*

**Figure 2:**
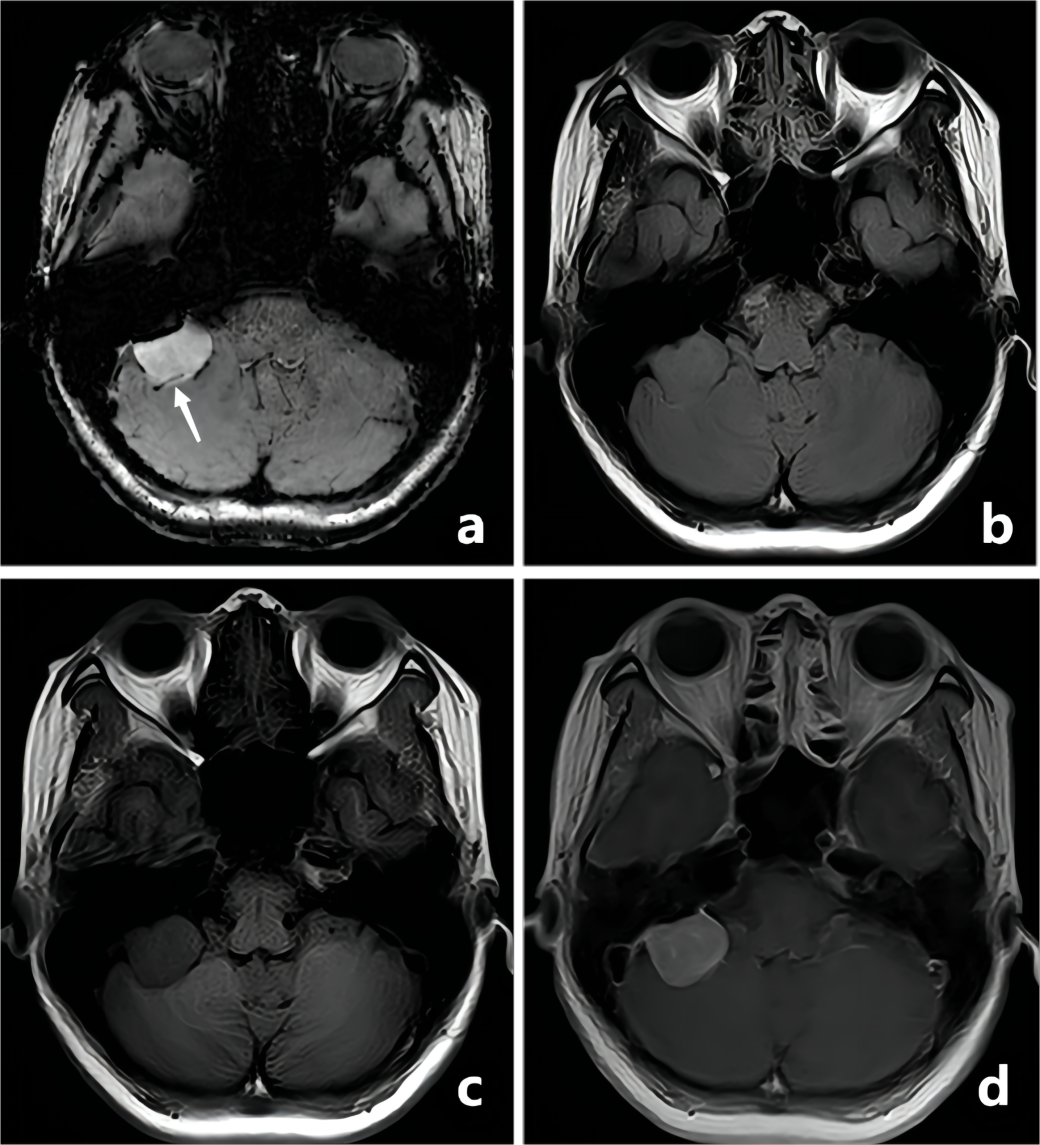
A-50-year-old woman with a pathologically confirmed meningioma in her right cerebellopontine angle area after surgical resection. The meningioma showed obvious homogeneous enhancement, and the intratumoral blood supply vessel appeared as linear hypointensity on CE-T_1_WI FSBB (**A**, white arrow). FLAIR **(B)**, T_1_WI **(C)**, and CE-T_1_WI **(D)** showed no signs of blood vessels.

**Figure 3:**
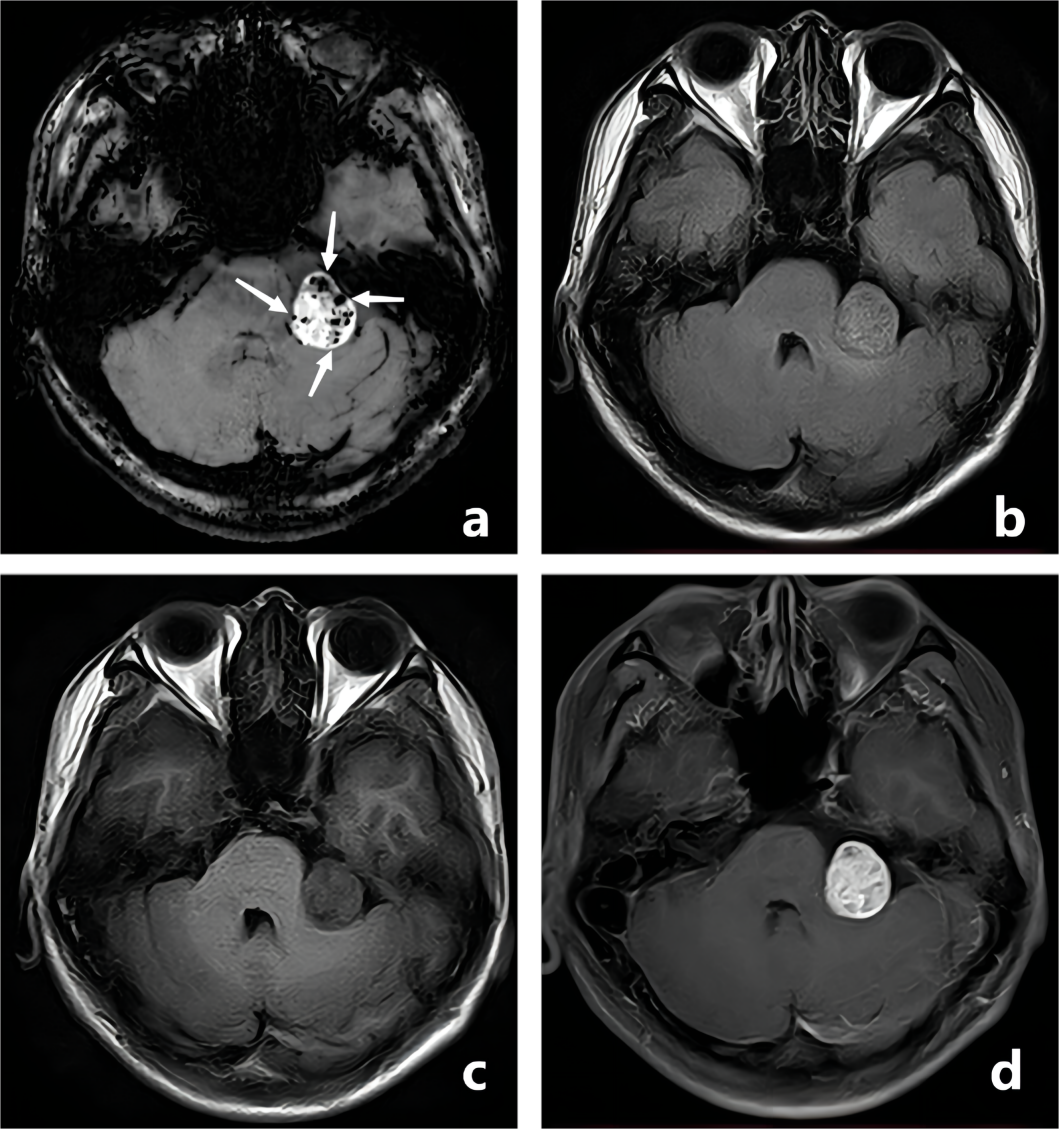
A-28-year-old man with a pathologically confirmed schwannoma in his left cerebellopontine angle area after surgical resection. The schwannoma showed obvious inhomogeneous enhancement, and the intratumoral microbleeds showed speckled low signals on CE-T_1_WI FSBB (**A**, white arrows). Microbleeds were not clearly visible on FLAIR **(B)**, T_1_WI **(C)**, and CE-T_1_WI **(D)**.

All CE-T_1_WI FSBB images were independently reviewed by two neuroradiologists who were blinded to each other, using the same picture archiving and communication system. Reader 1 (XC) and Reader 2 (KL) had 5 and 10 years of experience in diagnosing brain tumors by MRI, respectively. Images whose quality did not fulfill the required standards were excluded after unanimous consent from both neuroradiologists. They counted the intratumoral vessels and microbleeds based on the typical imaging features: (I) the intratumoral vessels showed linear hypointensity on CE-T_1_WI FSBB (**Figure 2**), and (II) the intratumoral microbleeds displayed speckled low signals on CE-T_1_WI FSBB (**Figure 3**), generally ranging in diameter from 2.0 to 5.0 mm and up to 10.0 mm. However, it was necessary to differentiate calcification and hemorrhage with brain CT examination.

**Figure 4:**
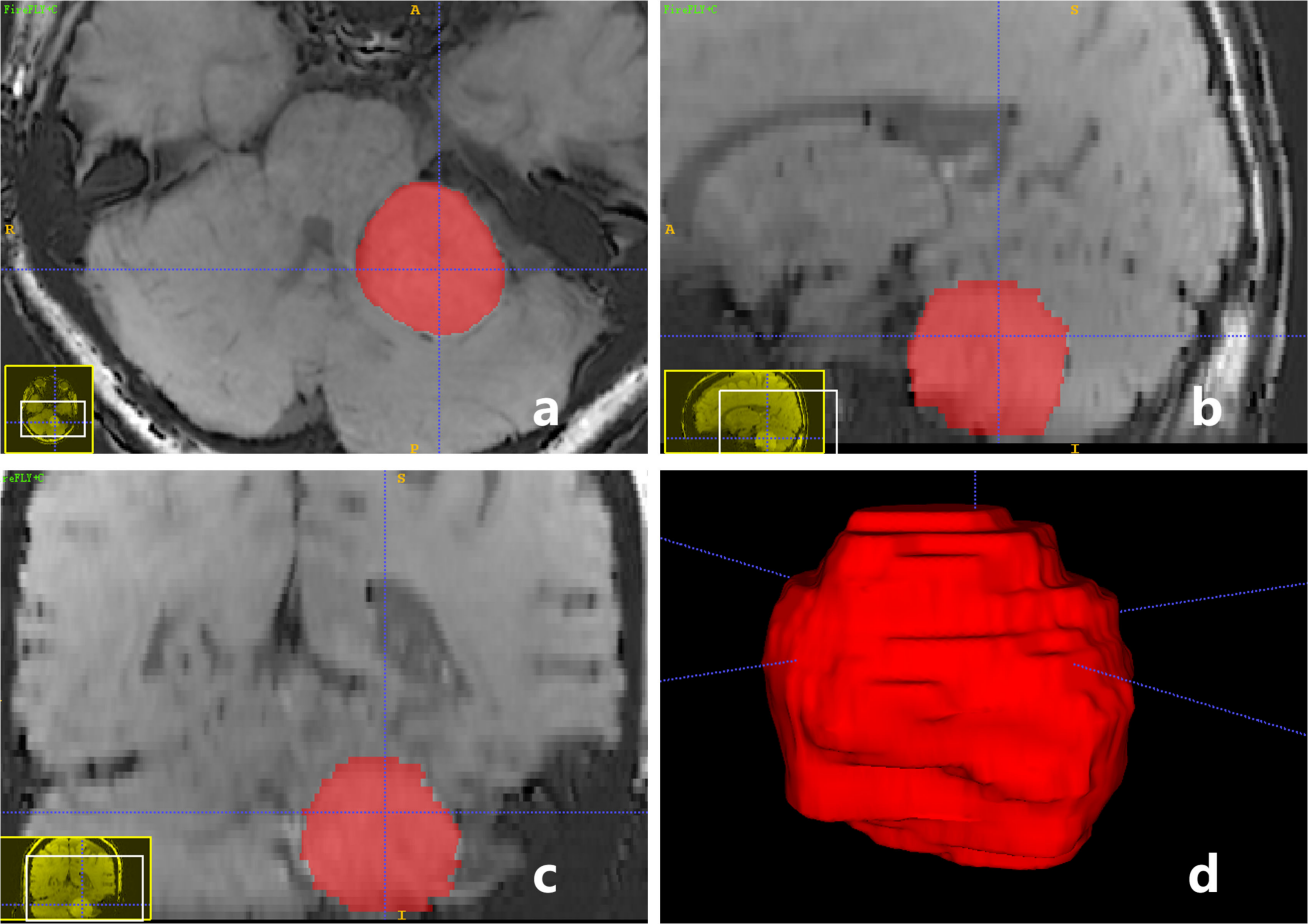
A-59-year-old woman with a pathologically confirmed meningioma in her left cerebellopontine angle area after surgical resection. ROI of tumor was drawn on CE-T1WI FSBB images slice-by-slice on the original axial **(A)**, reconstructed sagittal **(B)**, and reconstructed coronal **(C)** images, to obtain a 3D reconstructed volume of the tumor automatically **(D)**. Tumor volume = 16.95 (cm^3^), VDI = 0.27, MDI = 0.


ITK-SNAP software v.3.6.0 (created by Paul Yushkevich, Ph.D., the University of Pennsylvania, Pennsylvania, USA, and Guido Gerig, Ph.D., the University of Utah, Utah, USA, http://www.itksnap.org/pmwiki/pmwiki.php) was used for 3D manual segmentation and tumor volume calculation. ROI (region of interest) was drawn on all CE-T_1_WI FSBB images slice-by-slice on the original axial, reconstructed sagittal, and reconstructed coronal images (**Figures 4A-C**). We drew the maximum range of the lesion slightly along the visible boundary to cover the entire tumor volume, which included as few peripheral structures as possible. The delineation of the ROI was reviewed and approved by two neuroradiologists with more than 20 years of experience, they passed the consistency test, and the ICC was 0.92, indicating that the consistency was high. The volume of the tumor was automatically calculated using ITK-SNAP software (**Figure 4D**), then averaged the two neuroradiologists’ results. Furthermore, the vessel density index (VDI) and microbleeds density index (MDI) were calculated to evaluate the blood supply vessels and microbleeds of the tumor using the following formulas: 

(1) VDI=Intratumoral Vessels (n)Tumor Volume (cm^3) 

(2) MDI=Intratumoral Microbleeds (n)Tumor Volume (cm^3) 

### Statistical analysis

The consistency test of intratumoral vessel count and intratumoral microbleeds count between the two neuroradiologists based on the same image is summarized using 2-way random ICC (C, 1). Mann–Whitney U-test and chi-square test were used to determine significant differences between meningiomas and schwannomas and between fibrous meningiomas and epithelial meningiomas. All statistical analyses were performed using the SPSS software version 23.0 (SPSS IBM Corp., Armonk, New York, USA). *P*<0.05 was considered statistically significant.

## Results

### Tumors’ characteristics

In total, 116 cases were diagnosed as meningioma or schwannoma after postoperative histopathological examination, of which 19 cases with CE-T_1_WI FSBB images that were greatly affected by skull artifacts were excluded. Of the 97 cases that were ultimately included in the analysis, 73 were meningiomas, and 24 were schwannomas; 31 (31.96%) were males, and 66 (68.04%) were females with an average age of 52.37 ± 11.55 years (**Table 1**).

**Table 1:** Characteristics of patients included in the study.

Characteristics	Value
Number of patients (n)	97
Age (years)	52.37 ± 11.55
Gender (n)	M=31; F=66
Tumor types	Meningiomas=73; Schwannomas=24
Intratumoral vessels (Reader1, n)	3.24 ± 3.30
Intratumoral vessels (Reader2, n)	3.10 ± 3.07
Intratumoral microbleeds (Reader1, n)	13.20 ± 27.23
Intratumoral microbleeds (Reader2, n)	12.26 ± 25.75
Tumor Volume (cm^3^)	17.00 ± 20.69
VDI (n/cm^3^)	3.17
MDI (n/cm^3^)	0.99 ± 1.92

VDI, vessel density index; MDI, microbleed density index.

### Consistency analysis of CE-T1WI FSBB images

The ICC between Reader 1 and Reader 2 for intratumoral vessels count based on CE-T_1_WI FSBB was 0.89 (95%CI:0.85-0.93), and the ICC for intratumoral microbleeds count based on CE-T_1_WI FSBB was 0.99 (95%CI:0.99-0.99), which indicated an excellent agreement. The mean counts of intratumoral vessels and microbleeds were 3.17 ± 3.10 and 12.73 ± 26.44, respectively.

### Differences in quantitative features of CE-T_1_WI FSBB images between meningiomas and schwannomas

A comparison of 73 meningiomas and 24 schwannomas based on CE-T_1_WI FSBB images revealed differences in the number of intratumoral microbleeds (*P*<0.01) and MDI values (*P*<0.01) (**Table 2**). Hemorrhagic foci were detected in 23 of 24 (95.83%) schwannomas and 23 of 73 (31.50%) meningiomas. However, there were no differences in the number of intratumoral vessels (*P*=0.64), VDI values (*P*=0.17), or tumor volume (*P*=0.33). The clinical characteristics (age and sex) of the two groups of patients were not statistically different.

**Table 2:** Comparison of CE-T_1_WI FSBB features between meningiomas and schwannomas.

	Meningiomas (n=73)	Schwannomas (n=24)	*P*
Age (years)	55.00 (46.00, 62.50)	53.50 (37.25, 56.75)	0.20
Gender (Male, %)	24 (32.88%)	7 (29.17%)	0.74
Intratumoral vessels (n)	3.00 (1.00, 4.50)	2.00 (1.50, 4.50)	0.64
Intratumoral microbleeds (n)	0.00 (0.00, 2.00)	21.25 (10.75,61.38)	**
Tumor Volume (cm^3^)	8.77 (4.87, 23.37)	7.36 (4.95, 12.93)	0.33
VDI (n/cm^3^)	0.20 (0.07, 0.39)	0.29 (0.15, 0.59)	0.17
MDI (n/cm^3^)	0.00 (0.00, 0.13)	2.51 (1.17, 4.73)	**

VDI, vessel density index; MDI, microbleed density index.

**P < 0.01, *P < 0.05.

### Differences in quantitative features of CE-T_1_WI FSBB images between fibrous meningiomas and epithelial meningiomas

Among the 73 meningiomas, 38 (52.05%) were fibrous meningiomas, 22 (30.13%) were epithelial meningiomas, 4 (5.48%) were atypical meningiomas, 3 (4.11%) were mixed meningiomas, 2 (2.74%) were hemangiomatous meningiomas, 2 (2.74%) were secretory meningiomas, 1 (1.37%) was chordoid meningioma, and 1 (1.37%) was calcifying psammomatous meningioma. Comparison between fibrous meningiomas and epithelial meningiomas based on CE-T_1_WI FSBB images revealed differences in the number of intratumoral microbleeds (*P*<0.01), MDI values (*P*<0.01), and sex of patients (*P*<0.05) (**Table 3**). The odds ratios (OR) of microbleeds and male sex for diagnosing epithelial meningiomas rather than fibrous meningiomas were 9.53 and 4.43, respectively. There were no differences in the number of intratumoral vessels (*P*=0.97), VDI values (*P*=0.91), or tumor volume (*P*=0.71). The ages of the two groups of patients were not statistically different (*P*=0.94).

**Table 3:** Comparison of CE-T_1_WI FSBB features between fibrous meningiomas and epithelial meningiomas.

	Fibrous Meningiomas (n=38)	Epithelial Meningiomas (n=22)	*P*
Age (years)	55.00 (46.75, 59.00)	53.00 (45.50, 63.25)	0.94
Gender (Male, %)	7 (18.42%)	11 (50.00%)	*
Intratumoral vessels (n)	2.00 (1.00, 4.00)	1.50 (0.38, 5.75)	0.97
Intratumoral microbleeds (n)	0.00 (0.00, 0.00)	1.00 (0.00, 7.13)	**
Tumor Volume (cm^3^)	8.84 (4.88, 24.43)	7.48 (4.43, 25.36)	0.71
VDI (n/cm^3^)	0.18 (0.09, 0.31)	0.20 (0.01, 0.41)	0.91
MDI (n/cm^3^)	0.00 (0.00, 0.00)	0.12 (0.00, 0.59)	**

VDI, vessel density index; MDI, microbleed density index.

**P < 0.01, *P < 0.05.

## Discussion

FSBB is a vessel imaging technique that suppresses blood flow signals by adding an MPG pulse to the GRE sequence. SPGR is a strong gradient field applied at the end of the TR of each radio-frequency (RF) pulse, which artificially causes the magnetic field to be uneven and accelerates proton dephasing to eliminate the residual transverse magnetization vector after echo acquisition of the previous excitation RF pulse (2). At the next RF pulse excitation, the generated MRI signal (transverse magnetization vector) depends only on the recovery of the longitudinal magnetization vector, thereby producing the image contrast of T_1_WI. The MPG pulse is a bipolar gradient applied at the end of the excitation RF pulse and the front of the readout gradient (Gro) in three directions: slice selection gradient (Gss), phase encoding gradient (Gpe), and Gro (**Figure 1**). The areas in the positive and negative gradients are equal to the horizontal time axis, the scattered phases caused by the gradient of the stationary spins cancel each other and do not affect the signal. After experiencing an MPG pulse, the protons moving at different flow rates in the blood produce different phase deviations. The blood flow velocity and direction in small intracranial blood vessels are complex, and the flow velocity and flow direction of protons in imaging voxels vary, forming phase accumulations of different distributions, causing the voxel signal to weaken or disappear, finally resulting in black-blood contrast. Moreover, combined with the principle of the real-to-magnetic contrast ratio function, cosine filtering is used to adjust the polarity to enhance the true value of the black blood signal and further improve the contrast between peripheral microvessels and surrounding tissues (2).

The MPG pulse intensity is controlled by the TE; the longer the TE, the better the black-blood effect, but the heavier the susceptibility artifacts and the lower the signal-to-noise ratio (15). Hemoglobin, related substances, and iron metabolites in the blood can cause phase changes in local magnetic fields, particularly for differences in magnetic susceptibility between microbleeds and surrounding tissues (16, 17), which show speckled low signals in the enhanced tumor. For blood flow with a small vessel diameter and low flow velocity, a long TE (20.0 ms or 40.0 ms) can be used for data acquisition. Takeshi et al. used FSBB for the first time to evaluate the morphology of the lenticulostriate artery (4). Our study is the first to use CE-T_1_WI FSBB to quantify blood vessels and microbleeds in preoperative meningiomas and schwannomas. We found excellent consistency in both intratumoral vessel count (ICC=0.89, 95%CI:0.85–0.93) and intratumoral microbleeds count (ICC=0.99, 95%CI:0.99–0.99) based on CE-T_1_WI FSBB, which was better than the consistency assessment of FSBB in Sachi’s research (ICC=0.89, 95%CI:0.73–0.96) (1), which may be attributed to the use of contrast agents to make the contour of the enhanced tumor more defined. Through accurate assessment of blood vessels or variant vessels before tumor resection, preoperative lipiodol interventional embolization can be performed to reduce intraoperative bleeding and complications and improve the total resection rate (18). Furthermore, by accurately peeling off the blood vessels and tumors, some necessary vessels can be preserved, and additional damage to vessels can be reduced.

By comparing the CE-T_1_WI FSBB image features of meningiomas and schwannomas, we found that the schwannomas had more intratumoral microbleeds and higher MDI values. Previous studies reported a very low incidence of intratumoral hemorrhage in vestibular schwannomas (19), which may be limited because the original MRI technology is not sensitive enough to identify microbleeds, and advances in imaging suggest that intratumoral hemorrhage in schwannoma is far more common than previously believed. Thamburaj et al. used a T2*-weighted GRE sequence to assess intratumoral hemorrhage in vestibular schwannomas and found evidence of microbleeds in 15 of 16 cases (93.8%), which is similar to our result (95.83%). Histopathological studies have suggested that intratumoral fibrosis might represent indirect evidence of prior microbleeds, so almost all patients in their study showed signs of schwannoma bleeding (11). The etiology of schwannomas being more prone to hemorrhage than meningiomas remains unclear, but the visualization of intratumoral microbleeds was useful in the differentiation of schwannomas from other CPA masses, particularly meningiomas (20). Various risk factors have been suggested, including large tumor size, rapid tumor growth, the intratumoral genesis of abnormal thin-walled vasculature, increased tumor vascularity, and mixed Antoni A and B cellularity on histopathology (19). However, our study showed no significant difference in tumor size and the quantity and density of intratumoral vessels between schwannomas and meningiomas. Accurate preoperative differential diagnosis may influence prognosis because meningiomas have a higher chance of preserving hearing function and a higher incidence of recurrence than vestibular schwannomas. Apart from providing diagnostic assistance, the recognition of microbleeds may also play a significant role in assessing the biological behavior of schwannomas. Increased death rates and a higher incidence of hearing loss have been observed in vestibular schwannomas with intratumoral hemorrhage than in those without any hemorrhage (21, 22). We speculated that the process of continuous cystic changes in schwannomas is accompanied by repeated and multiple microhemorrhages events, and persistent consequent chronic inflammation reactions caused by hemorrhage destroy the tumor-nerve barrier, boosts peritumoral adhesion, and then causes adverse consequences. Surgery may be needed for hemorrhagic schwannomas, and smaller schwannomas without microbleeds can be treated with either gamma knife surgery or conservative management, especially in elderly patients.

Both fibrous meningiomas and epithelial meningiomas belong to the WHO I-grade benign tumor; however, by comparing their CE-T_1_WI FSBB features, we found that epithelial meningiomas had a higher incidence of intratumoral hemorrhage, more microbleeds, and higher MDI values. Previous studies have reported a low incidence of intratumoral hemorrhage in meningiomas (23), which was lower than the 31.50% reported in our study. Some scholars believe that meningioma hemorrhage is not associated with a particular histological subtype (24, 25); however, another study showed that an increased hemorrhage tendency was associated with fibrous meningiomas (26). These results are inconsistent with our findings, indicating that there is still no unanimous understanding of the differences in intratumoral hemorrhage among the different pathological types of meningiomas. It is necessary to further increase the sample size for research with the help of the new CE-T_1_WI FSBB technology combined with pathological analysis. Wang et al. identified undifferentiated vessels in hemorrhagic meningiomas as a factor involved in the hemorrhagic mechanism; neither differentiated vessels nor the total number of tumor vessels showed a significant difference between hemorrhagic and non-hemorrhagic meningiomas (27). Our study, with more participants, also found no association between the number of intratumoral vessels and number of intratumoral microbleeds. The proposed mechanisms of hemorrhage in meningiomas include rupture of weakened or unusual blood vessels, endothelial proliferation with secondary vascular occlusion, direct vascular invasion by tumor cells, intratumoral vasoactive substance release (e.g., histamine), concomitant vascular malformation or aneurysm, and direct meningioma fragmentation with bleeding (27). In a future study, with the increased sample size of other pathological types of meningiomas (such as hemangiomatous meningioma, mixed meningioma, atypical meningioma, anaplastic meningioma, etc.), further comparative analysis should be carried out by CE-T_1_WI FSBB sequence scan.

This study had some limitations. First, we only obtained pathological results of brain tumors without the exact pathological number of intratumoral vessels or microbleeds as the gold standard. Second, except for fibrous meningiomas and epithelial meningiomas, the number of other types of meningiomas was too limited to be included in the statistical analysis and comparison. Third, 15 meningiomas and four schwannomas were excluded because of serious artifacts caused by the skull near the lesion, and the unqualified rate of image quality was 16.37%, indicating that CE-T1WI FSBB was insufficient in the imaging of tumors near the skull. Fourth, assessing some sequence images may provide clear rationale to apply CE-T1WI FSBB in brain tumors, and we will carry out further related research in the future. Fifth, we will add the blinded radiologists review the preoperative imaging of these tumors using the characteristics generated to see how accurate these characteristics are at delineating between meningioma and schwannoma.

In conclusion, the consistency of intratumoral vessel and microbleed imaging on the CE-T_1_WI FSBB sequence is excellent. According to the CE-T_1_WI FSBB imaging features, schwannomas have a higher incidence of intratumoral hemorrhage, more intratumoral microbleeds, and higher MDI values than meningiomas, which aids in preoperative differential diagnosis and treatment decisions. Compared with fibrous meningiomas, epithelial meningiomas are more prone to imaging features of intratumoral microbleeds, which contributes to a better understanding of the biological behavior of different pathological types of meningiomas.

## Data availability statement

The raw data supporting the conclusions of this article will be made available by the authors, without undue reservation.

## Ethics statement

This study was approved by the Institutional Review Board of Huashan Hospital, Fudan University. Written informed consent was obtained from patients or their legal representatives before enrollment in the study. Written informed consent was obtained from the individual(s) for the publication of any potentially identifiable images or data included in this article.

## Author contributions

XC, KL, and SX share first authorship. Conceptualization: XC and KL. Methodology: SX. Software: XY. Resources: JZ. Data curation: ZF. Writing—original draft preparation: XC. Writing—review and editing: DG. Visualization: LP. Supervision: JZ. Project administration: XC. Funding acquisition: JZ. All authors have read and agreed to the published version of the manuscript. All authors contributed to the article and approved the submitted version.
